# A Coculture Model Mimicking the Tumor Microenvironment Unveils Mutual Interactions between Immune Cell Subtypes and the Human Seminoma Cell Line TCam-2

**DOI:** 10.3390/cells11050885

**Published:** 2022-03-04

**Authors:** Fabian A. Gayer, Alexander Fichtner, Tobias J. Legler, Holger M. Reichardt

**Affiliations:** 1 Institute for Cellular and Molecular Immunology, University Medical Center Göttingen, 37073 Göttingen, Germany; fabian.gayer@med.uni-goettingen.de; 2 Clinic of Urology, University Medical Center Göttingen, 37075 Göttingen, Germany; 3 Institute of Pathology, University Medical Center Göttingen, 37075 Göttingen, Germany; alexander.fichtner@med.uni-goettingen.de; 4 Department of Transfusion Medicine, University Medical Center Göttingen, 37075 Göttingen, Germany; tlegler@med.uni-goettingen.de

**Keywords:** testicular germ cell cancer, seminoma, Tcam-2, inflammation, T cells, monocytes, tumor microenvironment

## Abstract

Testicular germ cell cancer (TGCC) is the most common type of cancer in young men. Seminomas account for around half of them and are characterized by a pronounced infiltration of immune cells. So far, the impact of the tumor microenvironment (TME) on disease progression, especially the interaction of individual immune cell subtypes with the tumor cells, remains unclear. To address this question, we used an in vitro TME model involving the seminoma-derived cell line Tcam-2 and immune cell subsets purified from human peripheral blood. T cells and monocytes were strongly activated when individually cocultured with Tcam-2 cells as revealed by increased expression of activation markers and pro-inflammatory cytokines both on the mRNA and protein level. Importantly, the interaction between tumor and immune cells was mutual. Gene expression of pluripotency markers as well as markers of proliferation and cell cycle activity were upregulated in Tcam-2 cells in cocultures with T cells, whereas gene expression of *SOX17*, a marker for seminomas, was unaltered. Interestingly, the impact of monocytes on gene expression of Tcam-2 cells was less pronounced, indicating that the effects of individual immune cell subsets on tumor cells in the TME are highly specific. Collectively, our data indicate that seminoma cells induce immune cell activation and thereby generate a strong pro-inflammatory milieu, whereas T cells conversely increase the proliferation, metastatic potential, and stemness of tumor cells. Although the employed model does not fully mimic the physiological situation found in TGCC in vivo, it provides new insights potentially explaining the connection between inflammatory infiltrates in seminomas and their tendency to burn out and metastasize.

## 1. Introduction

Testicular germ cell cancer (TGCC) is the most common malignancy in young men [1]. Based on its histopathology, TGCC can be subdivided into seminomas and the more heterogeneous group of non-seminomas, both types with quite similar incidences but significant differences in treatment and prognosis [2,3,4,5]. In general, TGCC show extremely high cure rates as a consequence of primary surgery and a combination with effective platin-based chemotherapy in advanced stages of cancer. Seminomas are especially characterized by a pronounced inflammatory infiltrate consisting of lymphocytes and monocytes/macrophages [6,7,8,9,10]. The role of these tumor-infiltrating immune cells (TIICs) and the question of whether they contribute to the excellent overall survival (OS) of the patients is widely unknown. It is believed that the tumor microenvironment (TME), which is composed of various immune cell types, fibroblasts, and endothelial cells is responsible for the “burned-out” phenotype of seminomas or at least influences the clinical outcome [11,12]. In contrast to TGCC, the contribution of infiltrating immune cells in other cancer entities is more established. For instance, a prolonged OS is significantly associated with a higher degree of T-cell infiltration in patients with biliary tract cancer [13]. Furthermore, immune scores based on the analysis of infiltrating leukocytes are highly relevant in routine diagnostics and used as prognostic markers, e.g., for breast cancer, colorectal cancer, or melanoma [14,15,16].

Monocytes/macrophages and T cells constitute the majority of infiltrating immune cells in TGCC [7]. Macrophages are well known for their ability to commit to different phenotypes depending on the micromilieu [17]. Classically activated macrophages (M1) display pro-inflammatory characteristics while several subtypes of alternatively activated macrophages (M2) share a variety of anti-inflammatory activities [18]. Tumor-associated macrophages (TAMs) resemble the M2 phenotype, which promotes angiogenesis and tumor progression [19], but up to now, there is only scarce knowledge of their role in TGCC. They seem to have the capacity to lyse seminoma cells [20], and macrophage gene signatures were found to be increased in advanced tumor stages [6]. The impact of T-cell subsets in terms of prognosis and OS is well established for a variety of cancers [21,22,23,24,25], but their role in TGCC remains controversial, too. There is evidence that CD8^+^ cytotoxic T cells (CTL) are responsible for the immunological control of the tumor [26], and recent work further suggested a link between CTL infiltration and tumor cell apoptosis triggered by the release of cytolytic granules [27]. Accordingly, T cell-dependent immune response directed against cancer-testis antigens could be demonstrated in the peripheral blood of TGCC patients [28].

Since there is no TGCC animal model available to investigate the interaction between the immune system and the tumor, in vitro systems based on TGCC cell lines are currently the best option to address this question. The Tcam-2 cell line shares many characteristics with human seminoma cells [29], but depending on the microenvironment, they convert to a phenotype resembling embryonal carcinoma (EC) [30,31]. It was further reported that the TME in TGCC is pro-inflammatory in nature [32], and work performed in an in vitro model based on coculturing total peripheral blood mononuclear cell (PBMC) preparations with Tcam-2 cells revealed an immediate change in the cytokine milieu of the TME [33]. These experiments attested the pro-inflammatory cytokine IL-6 a major involvement in testis cancer [33], but the contribution of individual leukocyte subpopulations contained in PBMC is yet unknown.

While the great impact of immune cells on TGCC is undoubted, the interaction between immune cell subsets and germ cell cancer cells has not been delineated in detail. It is neither known whether tumor cells in TGCC exert similar or opposing effects on T cells and monocytes/macrophages nor whether immune cells mutually alter the characteristics of tumor cells as well. To address these issues, we performed coculture experiments with T cell and monocyte preparations sorted from the peripheral blood of different healthy donors. With this approach, we could obtain intriguing new insights into the immune-related mechanisms presumably contributing to the progression of seminomas in TGCC patients.

## 2. Materials and Methods

### 2.1. Cell Culture

Tcam-2 cells [34] were provided by Daniel Nettersheim (University of Düsseldorf, Düsseldorf, Germany) and authenticated by the Leibniz Institute DSMZ (Braunschweig, Germany). They were cultured in DMEM/Ham’s F-12 L-Glutamine medium (Capricorn Scientific, Ebsdorfergrund, Germany) with 10% FCS and 1% penicillin/ streptomycin at 37 °C and 5% CO_2_, maintained by splitting at a ratio of 1:3 every 5–6 days, and directly used without prior manipulation of the cell cycle stage.

### 2.2. Isolation of PBMC from Buffy Coats

Buffy coats were prepared from blood donations of anonymous healthy volunteers (14 in total). They were split into 15-milliliter aliquots, mixed with 15 mL of PBS/2% FCS/1 mM EDTA by gentle pipetting, and carefully layered on top of 15-milliliter Lymphoprep^TM^ (Stemcell Technologies, Cologne, Germany), thereby creating two separate phases. After centrifugation (800× *g*, 30 min, RT) and deceleration without brake, the turbid phase containing the PBMC was removed and washed once with PBS/2% FCS/1 mM EDTA. Cell counting was done under the microscope with the help of a Neubauer chamber.

### 2.3. Magnetic Cell Sorting

T cells or monocytes were isolated from 5 × 10^7^ PBMC either using the EasySep^TM^ human T cell Isolation Kit or the EasySep^TM^ Monocyte Enrichment Kit (Stemcell Technologies) according to the manufacturer’s instructions. The sorted immune cells were collected in 1 mL of growth medium and counted. To check their purity, a small aliquot of the cells was stained with an anti-hCD3 (T cells) or anti-hHLA-DR (monocytes) antibody and analyzed by flow cytometry. The purity was routinely >98%.

### 2.4. Coculture Experiments

1.5 × 10^5^ Tcam-2 cells were seeded in 2 mL of full growth medium in 6-well plates as previously described [33] and cultured for 24 h until they had fully adhered. Subsequently, sorted T cells (3 × 10^6^ cells/well) or monocytes (2 × 10^6^ cells/well) were added in a final volume of 4 mL, and cocultures were incubated at 37 °C and 5% CO_2_ for 24 or 48 h. Similar numbers of immune cells and Tcam-2 cells each cultured alone served as references. T cells or monocytes were harvested by gently resuspending them in the cell culture supernatant and aspirating them from the top of the Tcam-2 cell monolayer. After centrifugation (350× *g*, 7 min), the liquid phase and the cell pellet were separately stored for further analyses. Adherent Tcam-2 cells were harvested by adding 1 mL of pre-warmed 0.1% trypsin-EDTA (ThermoFisher, Waltham, MA, USA) for 1 min followed by repeated pipetting until a single cell suspension was achieved. The Tcam-2 cells were then washed with full growth medium and used for analysis. In general, 10% of each cell preparation was analyzed by flow cytometry, while RNA was prepared from the remaining 90%. Flow cytometric analysis revealed a purity of >98% for T cells, >90% for monocytes, >90% for Tcam-2 cells from T-cell cocultures, and >50% for Tcam-2 cells from monocyte cocultures.

### 2.5. Flow Cytometry

Cells were stained with different combinations of fluorochrome-conjugated monoclonal antibodies according to standard protocols [35]. All antibodies were obtained from BioLegend (Uithoorn, The Netherlands, clone names in brackets): anti-hCD69 (FN50), anti-hCD154 (24–31), anti-hCD25 (BC96), anti-hCD14 (HCD14), anti-hHLA-DR (I.243), anti-hCD3 (HIT3a), anti-hCD4 (OKT4), anti-hCD8 (HIT8a), and anti-hCD163 (GHI/61). Data were recorded with a FACS Canto II device (BD Bioscience, Heidelberg, Germany) and analyzed with FlowJo^®^ software (Tree Star, Ashland, OR, USA; version 10.7.0). Gating strategies are depicted in the respective figures.

### 2.6. Quantitative RT-PCR

Total RNA was prepared with the help of the Quick-RNA MiniPrep kit (Zymo Research, Irvine, CA, USA), and 1 µg each was reverse transcribed into cDNA using the iScript kit (Bio-Rad, Munich, Germany). Quantitative RT-PCR analysis was done on an ABI 7500 Instrument (ThermoFisher, Waltham, MA, USA) by employing the respective SYBR Green Master Mix. The housekeeping gene *18SRNA* was used for normalization, relative expression levels of target genes were calculated with the ΔΔCt method. All primers were synthesized by Metabion (Planegg, Germany), their sequences are listed in Table S1.

### 2.7. ELISA

Concentrations of IL-6 and TNFα in cell culture supernatants were determined using commercially available ELISA kits for the respective human cytokines (Biolegend, Uithoorn, The Netherlands according to the instructions of the manufacturer. Supernatants were prediluted 1:20 (IL-6) or 1:2 (TNFα) prior to the analysis. Data were recorded in a BioTek Power wave 340 Plate Reader (BioTek Instruments, Wetzlar, Germany).

### 2.8. Statistical Analysis

Data were analyzed either by unpaired *t*-test or one-way ANOVA followed by Newman-Keuls Multiple Comparison Test. All analyses were performed with GraphPad Prism^®^ software (San Diego, CA, USA; version 5.04). Data are depicted as scatter dots plots showing the mean as a horizontal line or as bar diagrams with the mean ± SEM. Levels of significance: *: *p* < 0.05; **: *p* < 0.01; ***: *p* < 0.001; n.s. (non-significant): *p* > 0.05.

## 3. Results

### 3.1. Tcam-2 Cells Induce T Cell Activation in a Coculture Model

**Figure 1:**
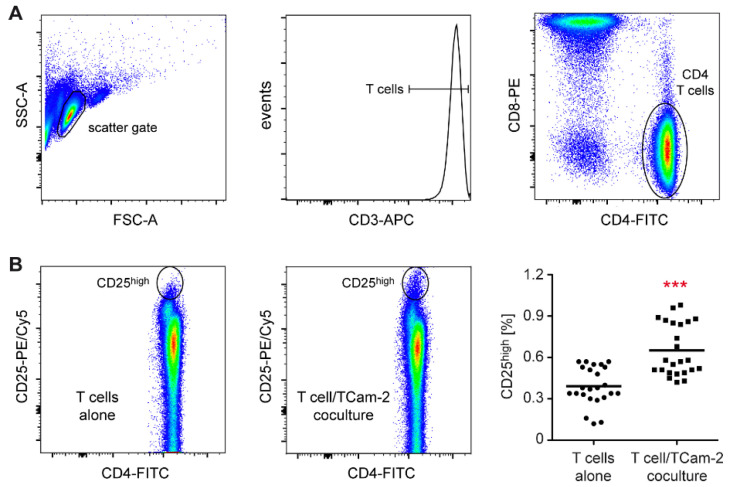
Flow cytometric analysis of peripheral blood T cells cocultured with TCam-2 cells. T cells were isolated from PBMC of healthy blood donors using magnetic cell sorting and cultured in 6-well plates either on a monolayer of TCam-2 cells or alone for 24 h. T cells were collected and stained with fluorochrome-conjugated monoclonal antibodies. (**A**) Exemplary gating strategy for T cell analysis. Relevant cells were initially defined based on their forward and sideward scatters, followed by gating for T cells based on the expression of CD3. T-cell subpopulations were further defined using the cell surface markers CD4 and CD8. (**B**) Activated CD4^+^ T cells were identified by their high expression of CD25. Exemplary comparison between T cells cultured alone (left panel) and T cells cocultured with TCam-2 cells (middle panel). The right panel depicts the quantification of CD25^high^ CD3^+^CD4^+^ T cells present in each condition. N = 24; PBMC were obtained from 7 healthy volunteers. Statistical analysis was performed by unpaired *t*-test (***: *p* < 0.001).

Tumor cells are well known for their ability to induce an immunosuppressive TME, thereby promoting the proliferation of neoplastic cells [36]. However, it has also been found that immune cells that get in contact with tumor cells become activated and initiate an inflammatory response [37]. To address this issue in an in vitro model, we first characterized changes in T cell activity after contact with the seminoma-like Tcam-2 cell line. T cells were sorted from the peripheral blood of healthy donors and cocultured for 24 h with Tcam-2 cells. T cells cultured alone served as a control. Importantly, flow cytometric analysis of the CD3^+^CD4^+^ T cell subset revealed an increased percentage of CD25^high^ cells with an activated phenotype in cocultures compared to T cells cultured alone (Figure 1).

**Figure 2:**
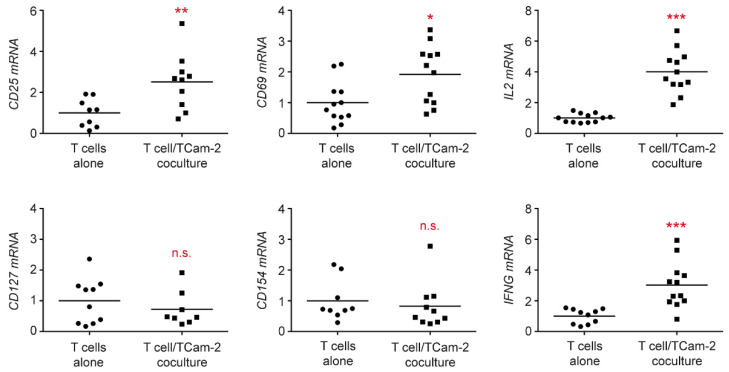
Gene expression analysis of peripheral blood T cells cocultured with TCam-2 cells by quantitative RT-QPCR. T cells were isolated from PBMC of healthy blood donors using magnetic cell sorting and cultured in 6-well plates either on a monolayer of TCam-2 cells or alone for 24 h. T-cells were collected from both cultures and RNA was prepared for subsequent analysis by quantitative RT-PCR. Relative mRNA expression levels of *CD25*, *CD127*, *CD69*, *CD154*, *IL2,* and *IFNG* are depicted as dot plots with a horizontal line representing the mean. Gene expression was calculated by normalization to the housekeeping gene *18SRNA*, mRNA levels in T cells cultured alone were arbitrarily set to 1. N = 8–12; PBMC were obtained from 5–6 healthy volunteers. Statistical analysis was performed by unpaired *t*-test (*: *p* < 0.05; **: *p* < 0.01; ***: *p* < 0.001; n.s.: *p* > 0.05).

To confirm our observation, a gene expression analysis was performed. Importantly, increased *CD25* mRNA levels were detected in cocultured T cells using quantitative RT-PCR whereas *CD127* expression in T cells was similar, indicating that Treg cells were unaltered in the presence of TCam-2 cells (Figure 2). To further corroborate T cell stimulation, *CD69* and *CD154* expression were analyzed. Interestingly, the early activation marker *CD69* was significantly upregulated while the late activation marker *CD154* was unaffected (Figure 2). Finally, we could demonstrate that gene expression of *IL2* and *IFNG* was strongly enhanced in T cells cocultured with Tcam-2 cells, reflecting an induction of proliferation and effector cell differentiation (Figure 2). Collectively, our data reveal that Tcam-2 cells induce T cell activation thereby promoting a pro-inflammatory TME.

### 3.2. Monocytes Cocultured with Tcam-2 Cells Acquire a Pro-Inflammatory M1 Phenotype

**Figure 3:**
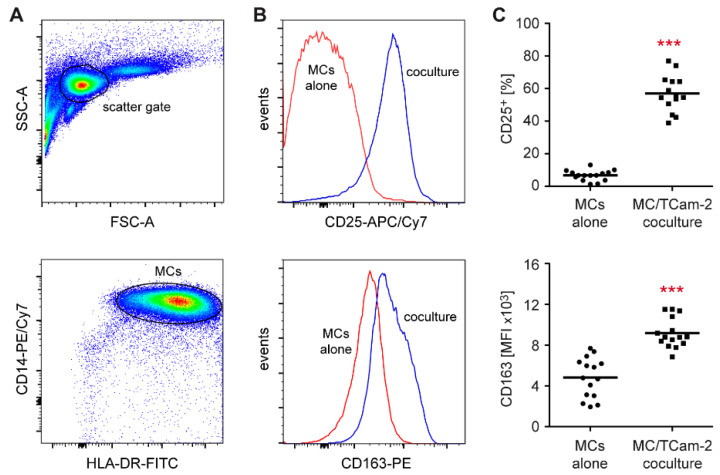
Flow cytometric analysis of peripheral blood monocytes cocultured with TCam-2 cells. Monocytes (MCs) were isolated from PBMC of healthy blood donors using magnetic cell sorting and cultured in 6-well plates either on a monolayer of TCam-2 cells or alone for 24 h. MCs were collected and stained with fluorochrome-conjugated monoclonal antibodies. (**A**) Exemplary gating strategy for MC analysis. Relevant cells were first identified on the basis of their forward and sideward scatters, followed by gating for MCs based on their co-expression of CD14 and HLA-DR. (**B**) Histograms of CD25 (upper panel) and CD163 (lower panel) surface expression on MCs either cultured alone (red line) and cocultured with TCam-2 cells (blue line). (**C**) Quantification of the percentage of CD25^+^ MCs (upper panel) or the MFI of CD163 (lower panel) in each condition. N = 14–15; PBMC were obtained from 5 healthy volunteers. Statistical analysis was performed by unpaired *t*-test (***: *p* < 0.001).

**Figure 4:**
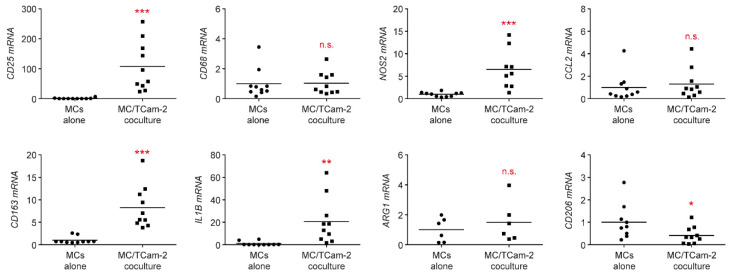
Gene expression analysis of peripheral blood monocytes cocultured with TCam-2 cells by quantitative RT-QPCR. Monocytes (MCs) were isolated from PBMC of healthy blood donors using magnetic cell sorting and cultured in 6-well plates either on a monolayer of TCam-2 cells or alone for 24 h. MCs were collected from both cultures and RNA was prepared for subsequent analysis by quantitative RT-PCR. Relative mRNA levels of *CD25*, *CD163*, *CD68*, *IL1B*, *NOS2*, *ARG1, CCL2,* and *CD206* are depicted as dot plots with a horizontal line representing the mean. Gene expression was calculated by normalization to the housekeeping gene *18SRNA*, mRNA levels in MCs cultured alone were arbitrarily set to 1. N = 6–10; PBMC were obtained from 4–5 healthy volunteers. Statistical analysis was performed by unpaired *t*-test (*: *p* < 0.05; **: *p* < 0.01; ***: *p* < 0.001; n.s.: *p* > 0.05).

In general, monocytes in the TME have the tendency to adopt an anti-inflammatory M2 phenotype and differentiate into so-called tumor-associated macrophages (TAMs) [38]. To characterize the ability of TGCC cells to reprogram monocytes in the TME, coculture experiments were performed. Flow cytometric analysis revealed strongly increased CD25 and CD163 surface levels on monocytes cocultured for 24 h with Tcam-2 cells, indicating an activated phenotype (Figure 3). In line with this finding, mRNA levels of *CD25* and *CD163* were dramatically induced in cocultures compared to monocytes cultured alone (Figure 4). Importantly, monocyte numbers presumably remained constant under both conditions as suggested by the unaltered gene expression of the pan-monocyte marker *CD68* (Figure 4). Further analysis revealed that the pro-inflammatory M1 markers *IL1B* and *NOS2* were strongly upregulated in cocultured monocytes whereas the two anti-inflammatory M2 markers *ARG1* and *CD206* were unaltered or even downregulated (Figure 4). No changes in gene expression were detected for the chemokine *CCL2* (Figure 4). In summary, our findings suggest that Tcam-2 cells potently induce monocyte activation and polarization to the pro-inflammatory M1 phenotype.

### 3.3. Increased Cytokine Production in the Tcam-2 Coculture Model

**Figure 5:**
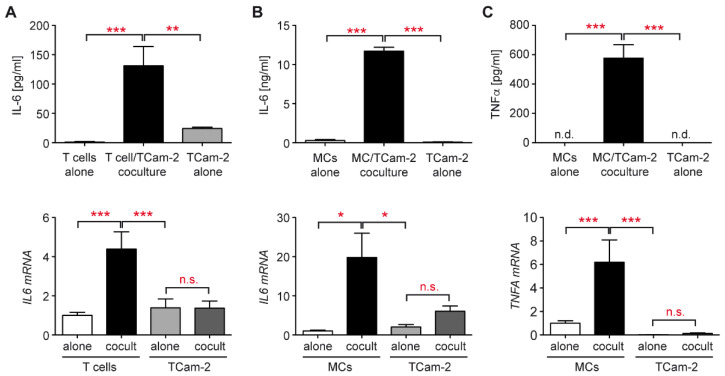
Analysis of cytokine secretion and release in immune cell cocultures with TCam-2 cells. T cells or monocytes (MCs) were isolated from PBMC of healthy blood donors using magnetic cell sorting and cultured in 6-well plates on a monolayer of TCam-2 cells for 24 h. T cells, MCs, and TCam-2 cells cultured alone served as controls. Supernatants were harvested for the analysis of secreted cytokines by ELISA, and the different cell types from each condition were collected for RNA preparation and subsequent analysis by quantitative RT-PCR. (**A**) IL-6 concentrations in the supernatant of T-cell cocultures and control monocultures were determined by ELISA (left panel, N = 10–13). *IL6* mRNA levels were calculated by normalization to the housekeeping gene *18SRNA* and arbitrarily set to 1 in T cells cultured alone (right panel, N = 7–10. (**B**) IL-6 concentrations in the supernatants of MC cocultures and control monocultures were determined by ELISA (left panel, N = 11–17). *IL6* mRNA levels were calculated by normalization to the housekeeping gene *18SRNA* and arbitrarily set to 1 in MCs cultured alone (right panel, N = 6–10). (**C**) TNFα concentrations in the supernatants of MC cocultures and control monocultures were determined by ELISA (left panel, N = 11–17). *TNFA* mRNA levels were calculated by normalization to the housekeeping gene *18SRNA* and arbitrarily set to 1 in MCs cultured alone (right panel, N = 10). All data are depicted as bar diagrams with the mean ± SEM. Statistical analysis was performed by one-way ANOVA and Newman-Keuls Multiple Comparison test (*: *p* < 0.05; **: *p* < 0.01; ***: *p* < 0.001; n.s.: *p* > 0.05). PBMC were obtained from 5 (ELISA) or 4–6 (quantitative RT-PCR) healthy volunteers, respectively.

Cytokines play central roles in tumor progression and metastasis. Previous work revealed a crucial involvement of IL-6 in testis cancer [33] and multifunctionality of TNFα in terms of apoptosis, cell survival, and inflammation [39,40]. To gain insights into the function of both cytokines in the interaction between Tcam-2 cells and individual immune cell subtypes, we performed coculture experiments and analyzed them by ELISA and quantitative RT- PCR. IL-6 secretion was detected in cocultures of T cells with Tcam-2 cells whereas T cells alone did not produce any IL-6 and Tcam-2 cells only very small amounts (Figure 5A). Gene expression analysis confirmed that T cells rather than Tcam-2 cells were the major source of IL-6 (Figure 5A). TNFα was undetectable in the supernatant of T cell cocultures and its gene expression was similar under all conditions (data not shown). Importantly, a massive secretion of IL-6 as well as TNFα was observed in cocultures of monocytes with Tcam-2 cells (Figure 5B,C), an effect which was much stronger than in cocultures with T cells. This indicates that the pro-inflammatory milieu established in response to immune cell activation by seminoma cells is dominated by monocyte-derived mediators. Gene expression analysis confirmed that both cytokines produced in cocultures mostly originate from activated monocytes rather than Tcam-2 cells (Figure 5B,C). Taken together, our data reveal that immune cells are stimulated by seminoma cells thereby presumably fostering pro-inflammatory conditions in the TME.

### 3.4. T Cells Promote a Phenotypic Transition of Seminoma Cells

**Figure 6:**
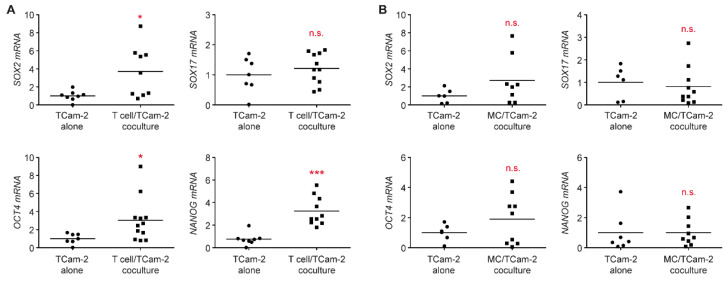
Analysis of gene expression of pluripotency markers in cocultures of TCam-2 cell with immune cells. T cells or monocytes (MCs) were isolated from PBMC of healthy blood donors using magnetic cell sorting and cultured in 6-well plates on a monolayer of TCam-2 cells for 24 h. TCam-2 cells cultured alone served as a control. TCam-2 cells were collected from both cultures, and RNA was prepared for subsequent analysis by quantitative RT-PCR. Relative mRNA levels of *SOX2*, *SOX17*, *OKT4,* and *NANOG* in TCam-2 cells collected from T cell cocultures (**A**), MC cocultures (**B**), or TCam-2 cells cultured alone are depicted as dot plots with a horizontal line representing the mean. Gene expression was calculated by normalization to the housekeeping gene *18SRNA*, mRNA levels in TCam-2 cells cultured alone were arbitrarily set to 1. N = 7–11; PBMC were obtained from 5–6 healthy volunteers (T cell cocultures); N = 6–10; PBMC were obtained from 4–5 healthy volunteers (MC cocultures). Statistical analysis was performed by unpaired *t*-test (*: *p* < 0.05; ***: *p* < 0.001; n.s.: *p* > 0.05).

Tcam-2 cells represent a cancer cell line reminiscent of seminomas [29,41]. The cellular plasticity and therefore the ability to undergo a transition to a divergent phenotype is well described for this tumor entity [31,42]. To investigate whether immune cells have an impact on phenotypic plasticity, we analyzed changes in gene expression in cocultured Tcam-2 cells. Significantly increased mRNA levels of the EC marker *SOX2* and the pluripotency markers *NANOG* and *OCT4* were observed in Tcam-2 cells cocultured with T cells compared to Tcam-2 cells cultured alone (Figure 6A). In contrast, mRNA levels of *SOX17,* which is a marker of seminoma cells, were similar in Tcam-2 cells regardless of the presence of T cells. Interestingly, changes induced by monocyte were much less pronounced and failed to reach significance (Figure 6B). This finding suggests that individual immune cell subsets impact the stemness of TGCC cells differentially and that T cells especially appear to mediate the transition from a seminoma to an EC phenotype.

### 3.5. The Proliferative Activity of Tcam-2 Cells Is Increased in T Cell Cocultures

**Figure 7:**
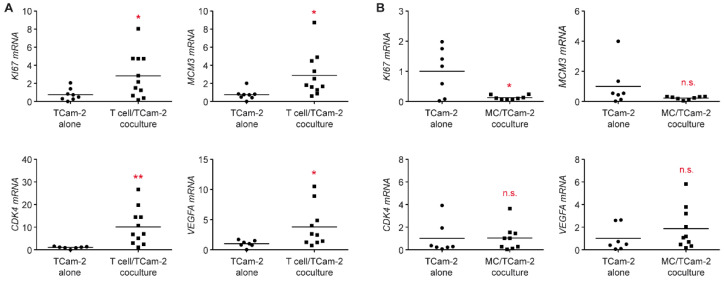
Analysis of gene expression of markers for proliferation and the metastatic potential in TCam-2 cell cocultures with immune cells. T cells or monocytes (MCs) were isolated from PBMC of healthy blood donors using magnetic cell sorting and cultured in 6-well plates on a monolayer of TCam-2 cells for 24 h. TCam-2 cells cultured alone served as a control. TCam-2 cells were collected from both cultures and RNA was prepared for subsequent analysis by quantitative RT-PCR. Relative mRNA levels of *KI67*, *MCM3*, *CDK4,* and *VEGFA* in TCam-2 cells collected from T cell cocultures (**A**), MC cocultures (**B**), or TCam-2 cells cultured alone are depicted as dot plots with a horizontal line representing the mean. Gene expression was calculated by normalization to the housekeeping gene *18SRNA*, mRNA levels in TCam-2 cells cultured alone were arbitrarily set to 1. N = 7–10; PBMC were obtained from 5–6 healthy volunteers (T cell cocultures); N = 7–8; PBMC were obtained from 4–5 healthy volunteers (MC cocultures). Statistical analysis was performed by unpaired *t*-test (*: *p* < 0.05; **: *p* < 0.01; n.s.: *p* > 0.05).

Considering that the TME is known to influence tumor behavior, we investigated key genes involved in proliferation and cell cycle activity. *KI67* and *MCM3* mRNA levels were significantly upregulated in Tcam-2 cells cocultured with T cells compared to Tcam-2 cells cultured alone, which indicates that T cells are presumably able to stimulate tumor growth (Figure 7A). Furthermore, mRNA levels of the cell cycle regulator *CDK4*, which is highly expressed in adult TGCC and therefore presents a therapeutic target [43], was strongly upregulated in Tcam-2 cells co-cultured with T cells (Figure 7A). Expression of *VEGFA*, a gene that has been associated with metastasis, was also increased in cocultured Tcam-2 cells, suggesting that T cells possibly increase the invasiveness of seminoma cells (Figure 7A). Again, monocytes affected Tcam-2 cell much less and even seemed to diminish proliferation (Figure 7B). Collectively, our data suggest that T cell infiltration in seminomas presumably promotes their proliferation and that the composition of immune cells in the TME thus might influence tumor outcome.

**Figure 8:**
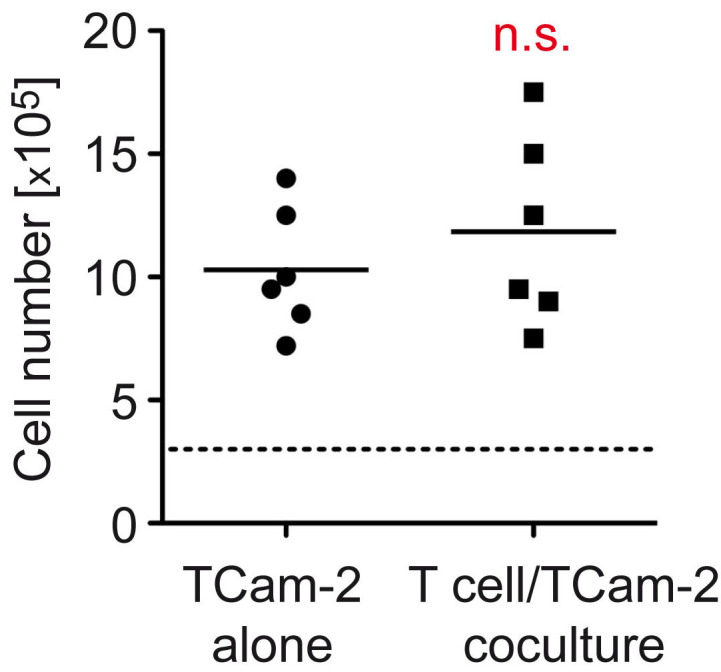
Analysis of TCam-2 cell growth in T cell cocultures. T cells isolated from PBMC of healthy blood donors by magnetic cell sorting were cultured in 6-well plates on a monolayer of TCam-2 cells for 48 h. TCam-2 cells cultured alone served as a control. TCam-2 cells were collected after removal of T cells and counted using a Neubauer chamber. N = 6; PBMC were obtained from 2 healthy volunteers. The dashed line represents the number of cells before culture. Statistical analysis was performed by unpaired *t*-test (n.s.: *p* > 0.05).

Finally, we set out to test whether the observed differences in *KI67*, *MCM3,* and *CDK4* expression resulted in a measurable change in cell growth. To this end, TCam-2 cells were cultured for 48 h in the absence or presence of T cells, followed by cell counting. In line with the increased mRNA levels of marker genes of proliferative activity, the number of TCam-2 cells in cocultures with T cells was 15% higher compared to TCam-2 cells cultured alone, although statistical significance was missed due to the low number of replicates (Figure 8). As of yet, the observed increase in cell number only represents a trend, but it still supports our conclusion that TIICs impact tumor growth.

## 4. Discussion

The TGCC seminoma subtype is characterized by an exceptionally pronounced inflammatory infiltrate accompanied by an excellent outcome [6,7,8,9,10]. It is thus crucial to better understand the link between the activity of TIICs and the disease course and OS of patients with seminomatous tumors. Therefore, the aim of this study was to delineate interactions of T cells and monocytes/macrophages with seminoma cells to obtain new insights into the role of the immune system in TGCC growth and metastasis relevant for patients in vivo [7]. To address this issue, we established an in vitro model based on the coculture of the seminoma-derived Tcam-2 cell line with purified T cells or monocytes isolated from PBMC of healthy blood donors. It is noteworthy that the characteristics of Tcam-2 cells have been extensively explored and shown to represent a suitable in vitro seminoma model [29,31,33,41,42]. Although Tcam-2 cells are not identical to seminomas in vivo, solid analogies exist regarding the expression of stem cells markers such as *OCT4, NANOG, PLAP, KIT,* and *D2-40* [29,41]. Moreover, Tcam-2 cells show a robust seminoma-like fate in situ after transplantation into the testis of immunodeficient mice [44]. Hence, we used the coculture of immune cell subtypes with Tcam-2 cells to mimick the TME of seminomas and analyzed mRNA levels, cytokine secretion, and changes in the cellular phenotype. Hereby, we unveiled specific and mutual interactions between these cell types providing new insights into the pathomechanism of seminomatous TGCC.

In this study, we showed that T cells and monocytes become activated in Tcam-2 cell cocultures and consequently assume a pro-inflammatory state. In the case of T cells, this was reflected by an increased percentage of CD25^high^ CD3^+^CD4^+^ cells accompanied by an upregulation of *CD25*, the early activation marker *CD69*, and the cytokines *IL2* and *INFG*. In contrast, the late activation marker *CD154* and the Treg cell marker *CD127* were unaltered. IL-2 is a cytokine that is crucial for T-cell proliferation [45], and IFNγ is one of the most important effector molecules of T cells. In combination, these two cytokines presumably contribute to a strong pro-inflammatory milieu in the infiltrated tumor. In the case of monocytes, we also found clear evidence for their activation in response to Tcam-2 cells. CD25 and CD163 surface levels are well known to be upregulated after stimulation of monocytes as is the case for the corresponding mRNA expression. It is also noteworthy that activated monocytes can polarize to opposite phenotypes depending on the conditions encountered in the TME. Tcam-2 cells induced genes encoding cytokines produced by pro-inflammatory M1 monocytes like *IL1B, IL6, TNFA,* and *NOS2* whereas gene encoding markers of anti-inflammatory M2 monocytes such as *ARG1* and *CD206* were unaltered or even downregulated. This indicates that monocytes not only become activated after contact with seminoma cells but also polarize to the M1 phenotype. In this respect, our results are in line with previously published work describing TGCC as an immunological cancer entity dominated by a pro-inflammatory cytokine milieu [32,46]. In addition, our results also match findings suggesting that testis cancer antigens elicit spontaneous CD4^+^ and CD8^+^ T-cell responses [28]. Intriguingly, the scavenger receptor CD163 on monocytes was induced in our model albeit it is mostly associated with an anti-inflammatory M2 phenotype [47,48,49]. However, it is also known that CD163 expression is induced by pro-inflammatory cytokines such as IL-6 [50], glycolytic metabolites produced by proliferating tumor cells such as lactate [51], and hormones including glucocorticoids [52]. Hence, we hypothesize that CD163 levels in monocytes are stimulated by compounds present in the TME rather than being the consequence of M2 polarization.

At first glance, our results seemingly contradict work published by Klein et al. based on a similar coculture model where no relevant alterations in the expression of a variety of pro-inflammatory genes were reported [33]. However, these authors used unsorted PBMC containing T cells, monocytes as well as a number of other immune cells in their coculture experiments while we employed highly purified preparations of individual immune cell subtypes. Considering that T cells and monocytes exert partially opposing effects on Tcam-2 cell proliferation, it is conceivable that the effect of immune cell subtypes might have concealed each other, thus explaining the different outcomes of both studies.

Immune cells are the natural source of cytokines; nevertheless, tumor cells are able to produce IL-6 and TNFα as well [53,54,55,56]. In our experiments, it was evident that both cytokines were only produced in cocultures at significant amounts but not in any of the control monocultures. Our data additionally indicate that T cells and monocytes rather than Tcam-2 cells account for cytokine production. Importantly, *IL6* and *TNFA* gene expression in cocultured Tcam-2 cells can be explained by contaminating monocytes that firmly stick to the tumor cell monolayer and cannot be completely removed prior to RNA extraction.

Besides the observed effects of TCam-2 cells on immune cell subtypes, we wondered whether their interactions were mutual. In particular, we hypothesized that seminoma cells undergo a phenotypic transition leading to their further dedifferentiation. Indeed, we could show that *SOX2* was upregulated in Tcam-2 cells when cocultured with T cells. Previously published work identified *SOX2* as a key player for reprogramming Tcam-2 cells from a seminoma phenotype to a more EC-like fate [31,57]. In contrast, the typical seminoma marker *SOX17* was unaltered in cocultured Tcam-2 cells. Another indicator for an ongoing dedifferentiation process is the expression of pluripotency markers which are also detected in EC [58]. Accordingly, we found that *NANOG* and *OCT4* were upregulated in Tcam-2 cells cocultured with T cells, indicating an increased stemness. Noteworthy, a complex consisting of Oct4 and Sox2 has been described as governing a regulatory hierarchy of differentiation [46,59]. The observed effects on the phenotype of seminoma cells were much more pronounced in cocultures with T cells than monocytes, revealing immune cell subtype-specific influences on TGCC cells. Although somewhat speculative, our results suggest a model in which activated T cells in the TME induce a phenotypic transition of seminoma cells to a more dedifferentiated state resembling an EC-like fate.

Another aim of our study was to understand the role of infiltrating immune cells in the TME with regard to tumor growth since the clinical outcome of patients is a central endeavor in immunological cancer research. Analysis of genes characterizing tumor cell proliferation and cell cycle activity revealed an upregulation of *KI67, MCM3,* and *CDK4* in Tcam-2 cells cocultured with T cells. The increased gene expression was accompanied by a trend towards higher TCam-2 cell numbers in cocultures, which suggests that T cells indeed promote seminoma cell growth. To the best of our knowledge, no efforts besides immunohistochemistry have been made up to now to study the impact of TIICs on the proliferation of seminoma cells in tissue specimens or Tcam-2 cells in vitro. It is worth mentioning that these genes have been widely used in the analysis and routine diagnostics of other tumor entities like breast, bladder, or prostate cancer and represent accepted predictors of clinical outcome [60,61,62]. Metastasis is another crucial feature of tumors and has been shown to correlate with *VEGFA* gene expression [63,64,65]. Our finding that *VEGFA* mRNA levels are increased in Tcam-2 cells cocultured with T cells can thus be considered as a first hint that TIICs might increase the tendency of seminomas to metastasize. However, undoubtedly, further experiments are needed to support this conclusion. Intriguingly, monocytes tended to reduce tumor cell proliferation, suggesting that individual immune cell subtypes might influence features of seminoma cells in an opposite manner. Nevertheless, it is likely that the impact of T cells on the phenotype and behavior of Tcam-2 cells is dominant.

TCam-2 cells are an established in vitro model of the seminoma subtype of TGCC and have been employed in a large number of studies reported in the literature. Considering the lack of an animal model mimicking seminomas, studies in cell culture are a valuable approach. Nonetheless, in vitro data still require confirmation by in vivo studies using primary tumor specimens. Hence, it will be exciting to find out whether the differences in gene expression observed in our TCam-2 coculture experiments are also present in tumor specimens from patients, for which reason we are currently collecting the respective material to conduct this study in the future.

## 5. Conclusions

In this study, we characterized interactions of purified immune cell subtypes with seminoma cells in an in vitro coculture model. For the first time, we were able to demonstrate that seminoma cells are able to induce a strongly pro-inflammatory TME by activating T cells and monocytes. Conversely, proliferation and possibly also the metastatic potential of seminomas was influenced by T cells, which also triggered a phenotypic transition to a dedifferentiated EC-like fate. These results clearly demonstrate that individual immune cell subtypes exert selective effects in an in vitro model of the seminoma TME and decipher their specific roles in determining the behavior and fate of this tumor entity.

## Data Availability

All data generated or analyzed during this study are included in this published article and its supplementary information files.

## Supplementary Materials

The following supporting information can be downloaded at: https://www.mdpi.com/article/10.3390/cells11050885/s1, Table S1: Primer sequences.
