# Supplementary material for: A Coculture Model Mimicking the Tumor Microenvironment Unveils Mutual Interactions between Immune Cell Subtypes and the Human Seminoma Cell Line TCam-2
Source: Cells. 2022 Mar 4;11(5):885. doi: 10.3390/cells11050885 (PMC8909655; doi:10.3390/cells11050885)
Supplement: Supplementary file 1 [file cells-11-00885-s001.zip › cells-1602944-supplementary.pdf]

Table S1. Primer sequences

Gene	Forward primer	Reverse primer
18SRNA	*TCC AGG TCT TCA CGG AGC TTG TT*	*GGA TGT AAA GGA TGG AAA ATA CA*
ARG1	*GGA GTC ATC TGG GTG GAT G*	*GGC ACA TCG GGA ATC TTT CC*
CCL2	*CCA GGA CTG CCT GAG ACA AG*	*CAG AAG CAA ACA CAG CCA CC*
CD25	*GTG GTG GGG CAG ATG GTT TA*	*TTG TGA CGA GGC AGG AAG TC*
CD68	*TCA GCT TTG GAT TCA TGC AG*	*TTG TAC TCC ACC GCC ATG TA*
CD69	*ACG CAG GTA GAG AGG AAC AC*	*ACC CTG TAA CGT TGA ACC AGT*
CD127	*GAA GGT TGG AGA AAA GAG TC*	*CAA AAT GCT GAT GGT TAG TAA*
CD154	*TCC CCC GGT AGA TTC GAG AG*	*ATT GTT GCC CG AAG GTT TG*
CD163	*GCA GGT TCC TCA AGA GGA GAG AA*	*ATG GCC TCC TTT TCC ATT CCA*
CD206	*CGA TCC GAC CCT TCC TTG ACT*	*AGT ATG TCT CCG GTT CAT GCC*
CDK4	*ATG GCT ACC TCT CGA TAT GAG C*	*CAT TGG GGA CTC TCA CAC TCT*
IFNG	*CTG TAA CTG CCA GGA CCC AT*	*TCT GTC ACT CTC CTC TTT CCA*
IL1B	*AGC CAT GGC AGA AGT ACC TG*	*TGA AGC CCT TGC TGT AGT GG*
IL2	*TTT ACA TCG CCA AGA AGG CCA*	*GCA CTT CCT CCA GAG GTT TG*
IL6	*TCA ATA TTA GAG TCT CAA CCC CCA*	*TTC TCT TTC GTT CCC GGT GG*
KI67	*TCC TTT GGT GGG CAC CTA AGA CCT G*	*TGA TGG TTG AGG TCG TTC CTT GAT G*
MCM3	*CGA GAC CTA GAA AAT GGG AGC C*	*GCA GTG CAA AGC ACA TAC CGC A*
NANOG	*GAT TTG TGG GCC TGA AGA AA*	*AAG TGG GTT GTT TGC CTT TG*
NOS2	*CGC ATG ACC TTG GTG TTT G*	*CAT AGA CCT TGG GCT TGC C*
OCT4	*CGA AAG AGA AAG CGA ACC AG*	*GCC GGT TAC AGA ACC ACA CT*
SOX2	*ATG CAC CGC TAC GAC GTG A*	*CTT TTG CAC CCC TCC CAT T*
SOX17	*GGC GCA GCA GAA TCC AGA*	*CCA CGA CTT GCC CAG CAT*
TNFA	*GCC CAT GTT GTA GCA AAC CC*	*TAT CTC TCA GCT CCA CGC CA*
VEGFA	*AGG GCA GAA TCA TCA CGA AGT*	*AGG GTC TCG ATT GGA TGG CA*
